# Cell wall evolution and diversity

**DOI:** 10.3389/fpls.2012.00152

**Published:** 2012-07-06

**Authors:** Jonatan U. Fangel, Peter Ulvskov, J. P. Knox, Maria D. Mikkelsen, Jesper Harholt, Zoë A. Popper, William G.T. Willats

**Affiliations:** ^1^Department of Plant Biology and Biotechnology, Faculty of Life Sciences, University of Copenhagen, Frederiksberg,Denmark; ^2^Centre for Plant Sciences, Faculty of Biological Sciences, University of Leeds,Leeds, UK; ^3^School of Natural Sciences, National University of Ireland,Galway, Ireland

**Keywords:** biomechanics, carbohydrate microarrays, CAZy, diversity, monoclonal antibodies, evolution, glycome, plant cell wall

## Abstract

Plant cell walls display a considerable degree of diversity in their compositions and molecular architectures. In some cases the functional significance of a particular cell wall type appears to be easy to discern: secondary cells walls are often reinforced with lignin that provides durability; the thin cell walls of pollen tubes have particular compositions that enable their tip growth; lupin seed cell walls are characteristically thickened with galactan used as a storage polysaccharide. However, more frequently the evolutionary mechanisms and selection pressures that underpin cell wall diversity and evolution are unclear. For diverse green plants (chlorophytes and streptophytes) the rapidly increasing availability of transcriptome and genome data sets, the development of methods for cell wall analyses which require less material for analysis, and expansion of molecular probe sets, are providing new insights into the diversity and occurrence of cell wall polysaccharides and associated biosynthetic genes. Such research is important for refining our understanding of some of the fundamental processes that enabled plants to colonize land and to subsequently radiate so comprehensively. The study of cell wall structural diversity is also an important aspect of the industrial utilization of global polysaccharide bio-resources.

## INTRODUCTION

Plant cell walls are multifunctional polysaccharide-rich fibrous composites in which polymers interact to form load-bearing structures embedded in a polysaccharide matrix (Bacic et al., 1988; Fry, 2004). Cells in the growing parts of plants are bound by “primary walls” in which the load bearing function is performed primarily by cellulose microfibrils. Models of the plant cell wall typically depict the microfibrils cross-linked with hemicelluloses, including mannans, xylans, mixed-linkage glucans (MLG), and xyloglucans. This network is then further embedded in a matrix of pectic polysaccharides including homogalacturonan (HG), rhamnogalacturonan-I (RG-I), rhamnogalacturonan-II (RG-II), and xylogalacturonan (Fry, 2004; Mohnen, 2008; Caffall and Mohnen, 2009; Harholt et al., 2010). However, this conventional description of primary walls that emphasizes tethering glycans as indispensible “load-bearing” structures may need revising as discussed in Scheller and Ulvskov (2010). Primary cell walls establish the foundations for cell shape and resist the tensile forces exerted by turgor pressure. They must also be capable of controlled expansion to enable cell growth. In non-growing plant tissues, some cells are typically surrounded by “secondary walls” whose primary role is to resist compressive force and since cell expansion is not required, these walls are often reinforced with lignin (Hepler et al., 1970; Carpita and Gibeaut, 1993; Boerjan et al., 2003; Cosgrove, 2005). Although these descriptions serve to describe many plant cell walls in broad terms, they are generalizations and are primarily based on investigations of the cell walls of flowering plants. However, cell walls display remarkable diversity at many levels and their constituent polysaccharides differ in fine structure, relative abundance, and molecular associations (Burton et al., 2010). The vast complexity and heterogeneity of cell wall glycomes is the product of the cooperative activities of prodigious numbers of biosynthetic enzymes. It is clear from genome sequencing that hundreds of glycosyltransferases (GTs) catalyze the formation of glycosidic linkages in polysaccharides –- more than 50 for the pectic polymers alone (Scheible and Pauly, 2004; Mohnen, 2008; Yin et al., 2010; Dhugga, 2012). Most GTs act in the Golgi apparatus and their products are transported to cell walls in secretory vesicles. In contrast, cellulose- and callose synthases, and possibly the “D” class of cellulose synthase-like GTs, are embedded in the plasma membrane and their products are extruded directly into cell walls (Endler and Persson, 2011; Park et al., 2011). The large numbers of GT-encoding genes and their varied temporal and spatial expression profiles produce vast possibilities for cell wall variability. Further heterogeneity is generated by the availability of a wide range of activated sugar donors (Feingold and Avigad, 1980), methylation and acetylation, different enantiomer and the variety and number of possible glycosyl linkages as well as *in muro* modification of polysaccharides, e.g., by esterification/deesterification of pectins and transglycosylation between certain hemicelluloses (Fry et al., 2008; Burton et al., 2010). Collectively, these dynamic processes enable plants to generate cell walls that are exquisitely suited to prevailing functional requirements and that can respond to biotic and abiotic stresses as well as developmental cues (Sarkar et al., 2009; Sørensen et al., 2010).

### WHY STUDY CELL WALL DIVERSITY?

The study of cell wall glycomes across the plant kingdom is important for developing our understanding of cell wall structures and functions, for understanding cell wall and plant evolution, and for optimizing the utilization of the largest source of biomass on earth. Plants emerged onto land around 470 million years ago and have since colonized a large proportion of the Earth’s surface (Kenrick and Crane, 1997; Waters, 2003; Niklas and Kutschera, 2010). The transition to land was a pivotal event in the history of life which resulted in the formation of new habitats and ecosystems and had profound effects on atmospheric chemistry. Cell walls have played significant roles in these epochal evolutionary events but our current understanding of many aspects of cell wall structures and their evolution is limited (Niklas, 2004; Popper and Tuohy, 2010; Sørensen et al., 2010). Improving our understanding will contribute to a wider understanding of plant evolution and phylogenetic relationships and may provide knowledge about past environments and insight into how plants might respond to predicted climate change scenarios.

The study of cell wall evolution is based largely upon the surveying of cell wall diversity (Popper, 2008; Sørensen et al., 2010; Popper et al., 2011). Only by doing this is it possible to correlate changes in plant physiology, morphology or habit with corresponding innovations in cell wall biology. A study of cell wall diversity across the plant kingdom also has other benefits. Cell wall polysaccharides are an immensely valuable renewable bio-resource and have numerous industrial applications. Timber, fibers, paper, functional ingredients (e.g., pectins from flowering plants and alginates and carrageenans from algae), and nutraceuticals, and first and second generation biofuels are predominantly cell wall-based (Bacic et al., 1988; Willats et al., 2006; Pauly and Keegstra, 2010). In contrast to nucleotide sequences and proteins, polysaccharides cannot readily be synthesized and so must be sourced from nature. Currently we use only a minute fraction of the global cell wall glycome and a comprehensive inventory of available polysaccharides may reveal valuable new molecules and materials with novel uses. The analysis of diverse cell wall compositions and architectures might also provide inspiration for current efforts aimed at the targeted modification of cell walls, notably for energy crops. However, surveying of cell walls across the plant kingdom is a daunting undertaking which as described below, entails many significant challenges and requires a multi-disciplinary approach. This is in large part because polysaccharides are not directly encoded by genome sequence; multiple enzymes are required to synthesize the activated sugar residues, linkages and many wall components undergo extensive modifications including methylation, esterification/deesterification, and acetylation as well as the addition of single or blocks of sugar residues.

## CHALLENGES IN SURVEYING CELL WALL DIVERSITY

The specific genes and enzymes that lead to synthesis of specific cell wall components has yet to be fully elucidated. Furthermore, in the majority of cases several enzymes are required to synthesize a specific cell wall component which may additionally undergo subsequent modification *in muro*. Consequently we are not yet at the stage where it is possible to determine cell wall composition and diversity via a comparative genomics approach and much of the knowledge so far gleaned has relied on polymer analysis. One fundamental difficulty associated with this is that polysaccharides are not amenable to facile sequencing. Their structures can be determined by several well established chemical methods which have been developed and applied to cell wall studies over the last 50 or so years. Each method has both limitations and merits but they may be applied in concert to reveal and determine cell wall complexity and diversity. Few of the methods developed so far are amenable to high throughput screening, so wide surveys have to rely on partial characterization initially. Fourier Transform Infra-Red Spectroscopy (FTIR) requiring little sample preparation can be high throughput and is useful for determining differences in cell wall composition across samples but is rarely effective for precise compositional analysis as it does not yield sequence information (Mouille et al., 2003). Recently, methods based on carbohydrate microarrays probed with monoclonal antibodies (mAbs) and carbohydrate-binding modules (CBMs) with specificities for cell wall polysaccharide epitopes have been developed. This technology can enable analysis of the occurrence of 100 epitopes in 2–3 days and can reveal much about cell wall composition despite some limitations largely derived from the current, although increasing, availability of characterized mAbs and CBMs (Moller et al., 2007; Sørensen et al., 2009; Pattathil et al., 2010). Application of FTIR and carbohydrate microarrays can be used to indicate cell walls that have a composition that may merit further and detailed analysis because they appear to be significantly different from characterized cell walls. Thus, use of these methods facilitates the development of hypotheses regarding cell wall composition that can be further explored by more detailed analysis of a subset of the sample set. Polysaccharide gel electrophoresis (PACE) (Goubet et al., 2002), Oligosaccharide mass profiling (OLIMP; Lerouxel et al., 2002), paper chromatography, and thin layer chromatography (Fry, 2001) and related approaches are powerful tools for the next level operating on a subset of the original sample set. Glycosyl linkage analyses and NMR make up the final tier as these methods are time consuming and for NMR also quite insensitive. These techniques are thus unsuitable for wide scale sampling but are often indispensible for in-depth analysis of selected samples. Whatever method of analysis is chosen, sampling will always pose significant challenges. Given that it is not feasible to sample the walls of every extant plant then what plants and what organs or tissues should be chosen and is there sufficient tissue available? It seems reasonable to select species that are representative of taxonomic groups or morphotypes, but such prioritizations are not always straightforward. Once plants are chosen then one is faced with the further difficulty of properly sampling the individual cell walls within that plant. One option would be to simply homogenize the whole plant and extract as many cell wall polysaccharides as possible. This is feasible for say, small seedlings and microalgae, but not for woody species and trees. Clearly then, certain tissues, organs or developmental stages need to be selected – but on what basis? Equivalence can also be problematic because plants differ in the organs and tissues they have. Some have flowers and leaves, some do not. Additionally, altered growth conditions may affect the expression and structure of cell wall components within the same species (Iraki et al., 1989a,b,c). Such considerations are important because we know that some cell wall components can be very selectively distributed throughout a plant (see section Fine mapping of cell wall diversity and heterogeneity at the cellular and subcellular levels) and can easily be missed. Interpretation of various analyses is a further important challenge. Considerable caution is required considering the near impossibility of truly inclusive sampling. If positively identified by multiple methods then a particular polysaccharide can be regarded as “present,” but unless all parts of a plant have been sampled (including all developmental stages) then failure to identify a particular polysaccharide should be interpreted as the presence of that polysaccharide being “unknown” rather than “absent.” When investigating cell wall evolution it is also important to consider polysaccharides that may occur at such low levels that they may be regarded as functionally unimportant in other studies. For example, although glycosyl linkage and ICP-MS data suggest RG-II or RG-II-like oligosaccharide occurs at very low levels in avascular bryophytes, less than 1% of that in angiosperms (Matsunaga et al., 2004), its presence in these plants would nevertheless be significant in terms of the evolution of underlying biosynthetic mechanisms.

## A MULTI-LEVEL APPROACH TOWARDS UNDERSTANDING CELL WALL DIVERSITY AND EVOLUTION

**FIGURE 1:**
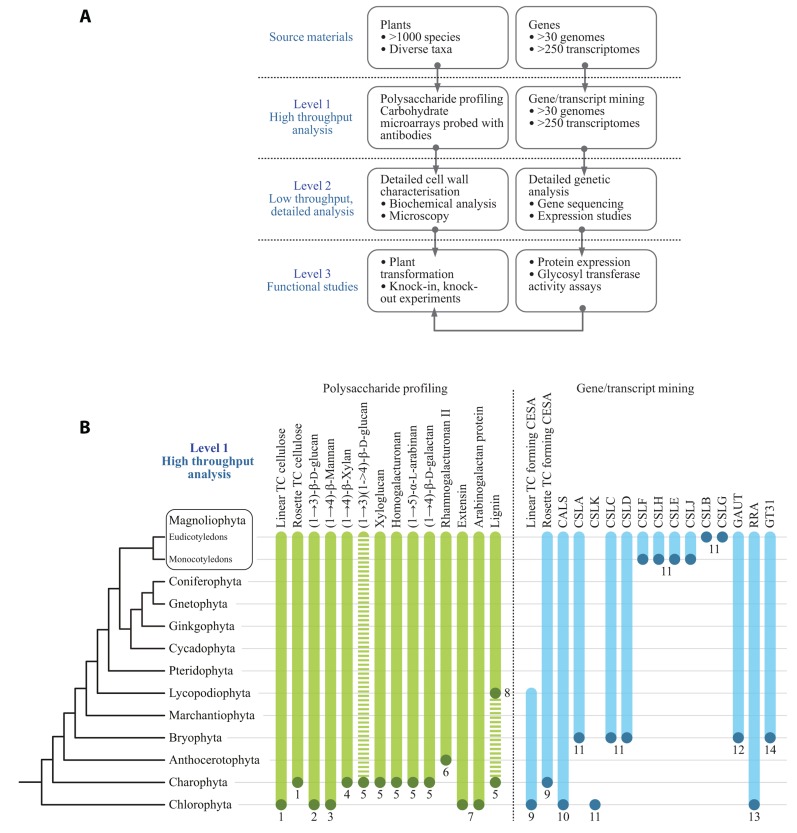
**(A)** A multi-level approach is required to assess the occurrence of cell wall polysaccharides and related genes throughout the plant kingdom. **(B)** Selected data from primary screens of polysaccharide occurrence in diverse plant species and results from mining genomes and transcriptomes for cell wall-related GTs. The dots at the bottom of the bars indicate the most basal plant group in which that particular polysaccharide or gene sequence has so far been identified. The bars indicate that it is assumed in most cases, once evolved, a gene or polysaccharide persists throughout evolution and is present in later diverging species. However, this is by no means universal. For example, the dashed bar for (1→3)(1→4)-β-D-glucan indicates that the occurrence of this polysaccharide is intermittent throughout the plant kingdom and has arisen by convergent evolution more than once. In some cases the presence of cell wall components is equivocal. For example, lignin has been tentatively identified in certain Charophycean green algae but the most basal group in which it has been definitively identified is the Lycopodiophyta. A clade of putative ancestral CSLs that are common ancestors to the *CSLA*s and *CSLC*s has been identified in certain Chlorophyte algae by Yin et al. (2009). These genes share homology with both *CSLA*s and *CSLC*s but appear to differ sufficiently to warrant a new family name, which we have assigned *CSLK*s, consistent with the existing alphabetical sequence of the existing *CSL* family names. (1) Tsekos (1999); (2) Scherp et al. (2001); (3) Mackie and Sto (1968); (4) Domozych et al. (2009); (5) Sørensen et al. (2011); (6) Matsunaga et al. (2004); (7) Estevez et al. (2009); (8) Weng et al. (2008); (9) Roberts et al. (2002); (10) Abercrombie et al. (2011); (11) Yin et al. (2009); (12) Yin et al. (2010); (13) Egelund et al. (2007), Velasquez et al. (2011); (14) Qu et al. (2004), Harholt et al. (2012). TC, terminal complex.

The authors have adopted a multi-level strategy for mapping cell wall polysaccharide and genetic diversity in order to gain insight into underlying evolutionary mechanisms (**Figure 1A**). The first level consists of primary screens for cell wall polysaccharides based on carbohydrate microarrays probed with mAbs and CBMs (Moller et al., 2007). This level is limited by the availability of characterized mAbs and CBMs and their ability to recognize the numerous epitopes which occur in the various plant cell wall components. Obviously the ideal would be to have as much coverage as possible of all the epitopes that exist within cell wall components. However, this is not the current situation and there are some notable wall components, such as RG-II, for which an effective mAb has yet to be generated. In parallel to carbohydrate microarrays, genomes and transcriptomes are mined to identify cell wall-related GTs. The second level of analysis seeks to obtain more detailed information about certain polysaccharides and genes from subsets of plants. These analyses are performed using established methods for polysaccharide analysis and gene cloning and sequencing. A third level is aimed at obtaining definitive information and the functions of genes, protein, and polysaccharides. In some cases genes are expressed and biochemical activities of GTs determined. **Figure 1B** shows some preliminary data from primary screens of polysaccharides and cell wall-related genes. The combined analysis of data sets can provide insight into the timing and mechanism of certain evolutionary events. For example, Xue and Fry (2012) have suggested that MLGs are restricted to horsetails based on the results obtained when diverse monilophyte cell walls were treated with lichenase, an enzyme that specifically fragments these (1→3)(1→4)-β-D-glucans. In contrast, we have obtained evidence for MLGs in *Selaginella moellendorffii* and selected Charophycean green algae (CGA) using microarray-based polysaccharide screening and lichenase treatments (Harholt et al., 2012; Fangel and Willats, unpublished)*. *Since a genome sequence is available for *S. moellendorffii* it is possible to establish with confidence that this plant does not contain orthologs of the *CSLF* and C*SLH *genes (Harholt et al., 2012) that are implicated in the synthesis of (1→3)(1→4)-β-D-glucan in Poales species (Burton et al., 2006; Doblin et al., 2009). These data provide good evidence that (1→3)(1→4)-β-D-glucan has evolved at least twice by convergent evolution.

### GENOME AND TRANSCRIPTOME MINING FOR CELL WALL-RELATED GLYCOSYL TRANSFERASES

“At least one GT for each glycosidic linkage” is axiomatic for our bioinformatic analysis of cell wall biogenesis. Rare dual function GTs are known from animals and plants, with CslA as an example of a GT that can utilize two different GDP-sugars as substrate. Accepting the axiom of one GT per glycosidic linkage should permit inference of the minimum number of GTs involved in cell wall biosynthesis. However, this is not possible for two reasons. Firstly, the evolution of flowering plants was accompanied by very substantial gene duplication. Differentiation of complex tissues in angiosperms calling for separate regulation in space and time of the same catalytic activity is a likely contributing factor. Mitchell et al. (2007) combined this line of thinking with expression analysis and proposed that certain clades of family GT61 should comprise genes involved in synthesis of a polymer of particular importance in grasses, a prediction that was recently proven correct (Anders et al., 2012). The large repertoire of GTs of the moss *Physcomitrella patens*, despite being non-vascular (so having non-lignified tissues only) and also having diverged prior to the gene duplication events associated with the evolution of flowering plants, cautions us not to generalize this principle excessively. The second reason why the number of GTs cannot be inferred from the number of different linkages is that the biosynthesis of some polysaccharides has turned out to be much more complicated than anticipated from the polysaccharide structure; xylan biosynthesis, reviewed by Scheller and Ulvskov (2010), provides a striking example. In dicots, GTs from two different families (GT43 and GT47) are implicated in synthesizing the xylan backbone totaling eight GTs for the synthesis of one linkage. Pectin biosynthesis is predicted to require at least 67 enzymes, GTs plus methyl- and acetyl transferases (Mohnen, 2008). Too few of these have been identified so far to judge whether Nature’s approach to pectin biosynthesis is lean, or expansive as with xylan. These limitations notwithstanding, it has proven fruitful to mine genomes for their GT repertoire. This is usually done using the CAZy database as a foundation, See **Box 1**. The CAZy database is the most extensive database of GTs and contains GTs from all three kingdoms. By using a global approach including the whole CAZy database in the screen, more remote orthologies can be discovered, exemplified by the discovery of a mannosylglycerate synthase of GT78 in *S. moellendorffii* (Scheller et al., 2010). But CAZy is not complete and GTs may be found outside CAZy and certain activities are hard to account for within the limit of the present CAZy database (Hansen et al., 2012). The CAZy driven approach has been applied to poplar (Geisler-Lee et al., 2006) and most recently to *Brachypodium distachyon* (Vogel et al., 2010) and *S. moellendorffii* (Banks et al., 2011) also leading to a CAZy-based naming convention for putative GTs that can be assigned to a gene family but not to a function.

**BOX 1 Text Box entitled CAZy.**

The CAZy database, www.cazy.org, describes the families of structurally related catalytic and carbohydrate-binding modules (or functional domains) of enzymes that degrade, modify, or create glycosidic bonds (Cantarel et al., 2009). GTs are classified to 94 families (and growing). The classification is partly based on sequence similarity, partly on 3D information of protein structure. The 94 families can be grouped into a small number of GT-folds creating a higher level in the hierarchy formalized as clans for glycoside hydrolases but not yet for GTs. Most families comprise several activities so assigning a GT to a family is rarely sufficient for deducing the precise catalytic activity of the GT. GTs may, however, with good precision be predicted to be inverting or retaining based on their CAZy family, where retaining refers to the situation where the transferred monosaccharide ends up in the same anomeric conformation as in the donor substrate and conversely for inverting GTs.

Applying the CAZy-based classification of putative GTs across large phylogenetic distances can yield evolutionary relevant information as exemplified by the case for convergent evolution of MLG. CAZy may also be employed as a support for gene discovery efforts as Egelund et al. (2007) did using homology between *Chlamydomonas* and *Arabidopsis* genes in clade A of family GT77 to infer the function of the Reduced Residual Arabinose (RRA) genes as encoding putative extensin arabinosyl transferases. This annotation is not proven but was corroborated by Velasquez et al. (2011). Extensin along with mannan are to the authors’ knowledge the only known shared cell wall components between Chlorophycean green algae and *Arabidopsis* (**Figure 1**; Estevez et al., 2009).

No CGA has yet been sequenced which is unfortunate given that cell wall analysis strongly suggests that the common ancestor of all plants, with xyloglucan and the pectic polymers typical of vascular plants, was a CGA (**Figure 1**). A number of EST datasets (Timme and Delwiche, 2010; Wodniok et al., 2011; Timme et al., 2012) are available and may be subjected to the same CAZy-based analysis as full genomes, albeit less safely. Our unpublished observations of this nature, in combination with recent phylogenetic analyses based on genomic data, lead us to propose that genera like *Penium* and *Coleochaete* represent the earliest versions of a higher plant cell wall while species like *Chara*, which superficially looks more advanced, has diverged substantially from the main evolutionary path leading to terrestrial plants and hence is a less attractive model for tracing the evolutionary history of the plants cell wall (Timme et al., 2012).

### FINE MAPPING OF CELL WALL DIVERSITY AND HETEROGENEITY AT THE CELLULAR AND SUBCELLULAR LEVELS

Cellulose is widely distributed in most cell walls but it is clear that the known structural diversity of the polysaccharides of the hemicellulose and pectic groups is regulated both taxonomically and also in relation to cell type and cell wall microstructures within tissues. The use of mAbs and CBMs for *in situ* analyses of cell walls remains a key approach to determine wall molecular architectures and their heterogeneities (Burton et al., 2010; Pattathil et al., 2010; Lee et al., 2011). The use of the same molecular probes in glycomic analysis is a very useful and complementary activity in which specific oligosaccharide structural features can be studied widely in terms of occurrence and biochemistry in addition to cellular locations.

**FIGURE 2:**
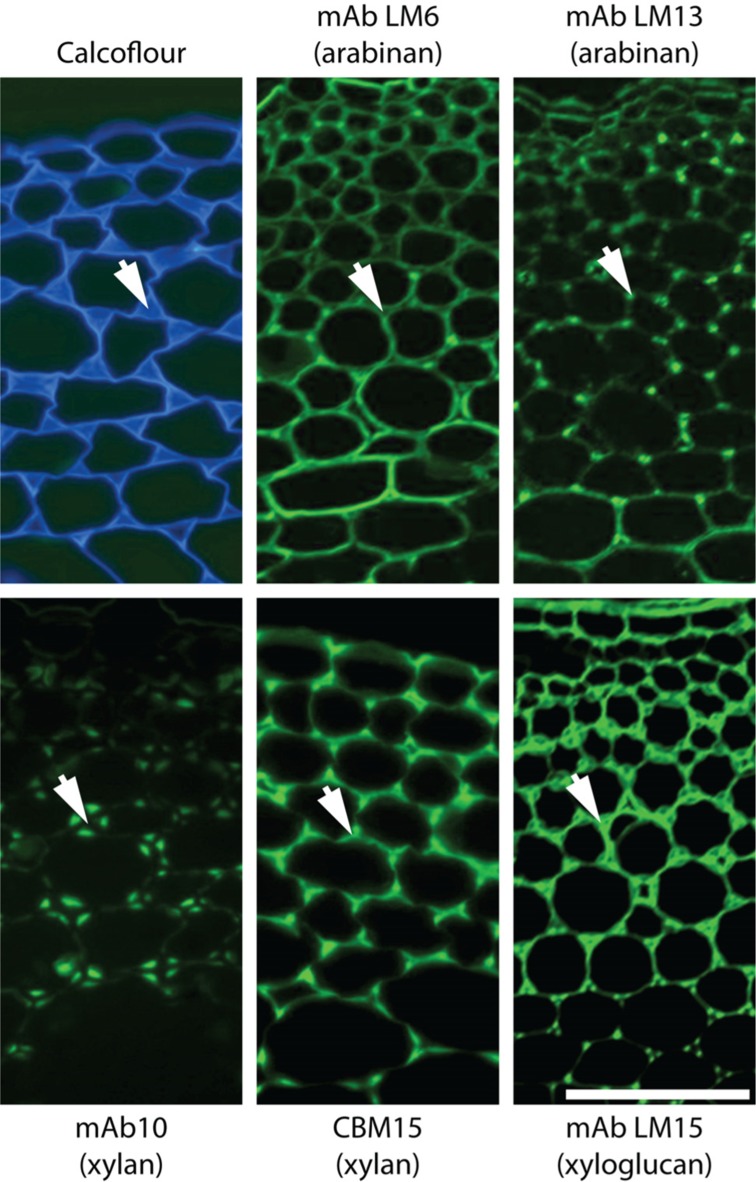
**Architectural heterogeneity in primary cell walls**. Equivalent transverse sections of the cortical region of tobacco stems with immunofluorescence imaging of two arabinan epitopes (mAbs LM6 and LM13), two xylan epitopes (mAb LM10 and CBM15) and one xyloglucan epitope (mAb LM15). Arrows indicate intercellular matrix shared by adjacent cells. Xylan and xyloglucan epitopes are shown after removal of pectic homogalacturonan. Pectic arabinan, xylan, and xyloglucan structural features all display spatial heterogeneity in relation to cell wall thickening and intercellular regions. mAb, monoclonal antibody; CBM, carbohydrate-binding module. Scale bar = 100 μm.

It is a significant point that although we know some broad cellular occurrences of specific non-cellulosic polysaccharides – such as the abundance of xylan in secondary cell walls of dicotyledons, of HG in primary cell walls and (an example from the subcellular level) the absence of pectic galactan from pit fields, we do not have a good understanding of the cellular distributions of all polymers for most plant cell types. Even for *Arabidopsis* a systematic *in situ* assessment of the major polymers in the cell walls of the major organs has not yet been achieved. Some can be predicted – such as those indicated above but the distributions of RG-I and other pectic epitopes or hemicelluloses cannot yet be predicted with certainty. *In situ* analyses of cell wall structures are made more complex but also more revealing in that as probe sets for specific polysaccharides are extended more cell wall diversity and heterogeneity can be uncovered. For example, this is the case for pectic HG (Willats et al., 2001; Parre and Geitmann, 2005; Wolf et al., 2009) and also for pectic arabinan (Verhertbruggen et al., 2009) where a mAb for linear arabinan detects arabinan substructures in restricted intercellular regions of parenchyma as shown in **Figure 2**. This also applies to xylan structures in the same parenchyma (Hervé, 2009). What is the functional basis for polysaccharide fine structure of both xylans and arabinans being so intimately spatially regulated in relation to factors such as cell adhesion (**Figure 2**)? Is this to be a paradigm for non-cellulosic wall polymers for which we currently have one or a limited number of probes such as pectic galactan and xyloglucan? The next few years will see more detailed systematic assessments of molecular architectures. This will be in conjunction with enzymatic and/or chemical pre-treatments that are in some cases required to optimize polysaccharide detection and in the case of polysaccharide masking (in which one polymer class blocks access to another polymer class) indicates important features of cell wall architectures reflecting protein access (Vreeland et al., 1984; Marcus et al., 2010; Davies et al., 2012).

Clearly we face many challenges in understanding cell wall evolution not least of which include sufficient sampling followed by appropriate synthesis and interpretation of large data sets including diverse information such as gene and protein sequence data as well as sugar linkages and epitope distributions. However, as cell and tissue molecular architectures are documented another major issue that is brought into focus is the function of individual wall components and of the entire cell walls, which can vary enormously with respect to quantitative and qualitative composition. Why do some cell walls have xyloglucan, xylan, and mannan hemicelluloses in distinct spatial distributions as for example in the extensively studied tobacco stem system as shown in **Figure 2**? How are these heterogeneities integrated into a functional whole in terms of wall properties and functions? *In vitro* analyses of composites formed from cellulose, pectins, and xyloglucans have yielded invaluable information about the properties of some wall components (Chanliaud et al., 2002). However, the structural and compositional complexity of naturally occurring cell walls means that an *in vitro* approach cannot reasonably be applied to investigate the functional properties of the full diversity of extant walls. *In vivo* methods of investigating wall biomechanics, at the tissue and lower levels, have been developed (Spatz et al., 1998; Burget, 2006) facilitating an improved understanding of how walls are assembled and the fine detail of wall domains and their nanomechanical properties are likely to emerge within the next few years as detailed *in situ* analyses are combined with genetic and enzymatic interventions. Integrating this knowledge will be a major challenge and is an exciting frontier for cell wall biology.

## Conflict of Interest Statement

The authors declare that the research was conducted in the absence of any commercial or financial relationships that could be construed as a potential conflict of interest.
